# Population based study of genital *Chlamydia trachomatis* prevalence and associated factors in Norway: A cross sectional study

**DOI:** 10.1186/1471-2334-12-150

**Published:** 2012-07-02

**Authors:** Hilde Kløvstad, Andrej Grjibovski, Preben Aavitsland

**Affiliations:** 1 Norwegian Institute of Public Health, PO box 4404, Nydalen, Oslo, 0403, Norway; 2 Norwegian Institute of Public Health, PO box 4404, Nydalen, Oslo, 0403, Norway; 3 Norwegian Institute of Public Health, PO box 4404, Nydalen, Oslo, 0403, Norway; 4 Current address: Epidemi, Aerfuglveien 53, Kristiansand, 4623, Norway

## Abstract

**Background:**

The number of diagnosed cases of *Chlamydia trachomatis* infection has been increasing in the past years in Norway although the testing rate has been relatively stable. The aim of this study was to measure the prevalence of genital *Chlamydia trachomatis* in young men and women in one county in Norway and determine associated factors in order to better target preventive measures.

**Methods:**

We mailed to a random sample of 10 000 persons aged 18–25 in Rogaland county a mail-back urine sample kit and a self-administered questionnaire with questions on socio-demographic details, health seeking behaviour and symptoms of and history of sexually transmitted diseases. Associations between current *Clamydia trachomatis* infection and the above mentioned factors were studied by multiple logistic regression.

**Results:**

The response rate among women was 18.9% (930/4923) and 11.9% (605/5077) among men. The prevalence of *Chlamydia trachomatis* infection was 5.8% (95% CI 4.5-6.8) among women and 5.1% (95% CI 3.8-6.8) among men. For men a greater number of partners during the last year (p for trend < 0.001), and living in a municipality without a local youth clinic increased the odds of infection (OR 8.6, 95% CI 2.2-33.9). For women a greater number of partners during the last year (p < 0.001) and not having consulted a family doctor for STIs (OR 2.1 95% CI 1.1-4.2) were positively associated with infection while not having a previous *Chlamydia trachomatis* diagnosis decreased the odds of having this infection (OR 0.3, 95% CI 0.2-0.7).

**Conclusion:**

Our results indicate the importance of having a visible youth clinic in each municipality. It also suggests targeting women who have had a previous *Chlamydia trachomatis* infection diagnosed before.

## Background

Control of genital *Chlamydia trachomatis* infection (hereafter: chlamydia) requires a range of activities, among those the promotion of safer sex and condom use, and early diagnosis and treatment [1]. Despite major efforts to increase the condom use and testing activity among young men and women in Norway, the testing rate has been relatively stable, 53 per 1000 population in 2000 and 51 per 1000 population in 2010, and the number of diagnosed chlamydia cases has been increasing[2,3]. The aim of this study was to measure the prevalence of chlamydia in men and women aged 18–25 years in Norway and determine associated factors in order to better target preventive measures.

## Methods

### Study design and study population

We conducted a cross sectional study among a random sample of 10 000 persons aged 18–25 years selected from the national population register with residence in one of Norway’s 19 counties, Rogaland (41 519 persons with birth year 1980–1987 in this area). This cross sectional study was part of a trial on the efficacy of home sampling for chlamydia *versus* no intervention in testing, diagnosing and treating chlamydia in this age group. We used the unique personal identity number of individuals living in Norway to randomize participants. An invitation was sent by mail to all10 000 with a mail back urine sample kit and a self-administered questionnaire. The urine sample kit consisted of information on chlamydia, an invitation to take a home test free of charge, a urine container, instructions on how to obtain a first void urine sample and a prepaid return envelope. The questionnaire included questions on socio-demographic details (age, sex, marital status, education, occupation, country of birth), health seeking behaviour, sexual behaviour (number of partners, contraception use at last sexual intercourse with steady and non-steady partner) and symptoms at the time of testing (discharge, endocervical bleeding, pelvic pain, urethral itching, dysuria) and history of sexually transmitted infections (STI). The participants were requested to send the urine sample by mail to the local hospital and the questionnaire to the Norwegian Institute of Public Health within three months after invitation. The study period lasted for three months in the period February- May in 2006.

### Information on outcomes

A participant was defined as a chlamydia case if the urine test was positive for chlamydia within the study period. All samples were analyzed with BD ProbeTec Chlamydia Amplified DNA assay (Becton, Dickinson, Franklin Lake, New Jersey, USA) [4-6].

### Statistical analysis

Confidence intervals (CI) for the prevalence of Chlamydia infection were calculated using Wilson’s method. Pearson’s chi-squared tests were used for bivariate analyses of nominal data. Interval data were compared using Student’s t-tests for unpaired samples.

Independent associations between the studied factors and the odds of having chlamydia were studied in multiple logistic regression. Crude and adjusted odds ratios (OR) were calculated.

The strongest predictors were identified using backward elimination procedure in multiple logistic regression. The selection of variables was based on the results of the likelihood ratio tests.

All analyses were performed using SPSS 14.0 (SPSS Inc., Chicago, IL).

### Ethics

The research project was approved by the Regional Committee for Medical and Health Research Ethics (REK), South-East region, in January 2006.

## Results

A total number of 1670 returned the questionnaire and submitted a urine sample for chlamydia testing during the study period, response rate 16,7%. Among those, we included the 1568 that were at risk, *i.e.* reported ever having had sexual intercourse. Due to missing values only 1535 were included. Altogether 930 (60%) of the respondents were women, response rate 18,9% . Among men the response rate was 11.9% (605/5077). The response rate was higher in the age group 22–25 years (16.6%, 828/4995) than in the age group 18–21 years (14.1%, 707/5005). The mean age was 22.0 (SD = 2.2) years for men and 21.6 (SD = 2.2) years for women (t _(1533)_ = 3.44, p < 0.001).) Only 5.6% of the participants were non-Norwegians, with continents of birth equally distributed between Europe/North America and other continents (Asia, South and Central America, Africa and Oceania) (Table 1).

**Table 1:** Prevalence of genital chlamydia infection and socio-demographic factors and health care utilisation, sexual behaviour and self-reported STIs among men and women aged 18–25 years in Rogaland, Norway. N = 1535

		**Men (n = 605)**			**Women (n = 930)**		
		**n/N**	**%**	**95% CI**	**n/N**	**%**	**95% CI**
**Age**	18-21	16/254	6.3	3.9-10.0	30/453	6.6	4.7-9.3
	22-25	15/351	4.3	2.6-6.9	24/477	5.1	3.4-7.4
**Marital status**	Single	30/550	5.5	3.8-7.7	54/817	6.6	5.1-8.5
	Married	1/55	1.8	0.3-9.6	0/113	0	0-3.2
**Education, years**	≤14	29/490	5.9	4.2-8.4	45/675	6.7	5.0-8.8
	15+	2/115	1.7	0.5-6.1	9/255	3.5	1.9-6.6
**Occupation**	Employed	18/339	5.3	3.4-8.2	23/336	6.8	4.6-10.1
	Unemployed	4/27	14.8	5.9-32.5	4/37	10.8	4.3-24.7
	Student/pupil	8/197	4.1	2.7-7.8	20/479	4.2	2.7-6.4
	Other/Unknown	1/42	2.4	0.4-12.3	7/78	9.0	4.4-17.4
**Country of birth**	Norway	28/575	4.9	3.4-7.0	52/876	5.9	4.6-7.7
	Europe/North America	0/13	0	0-22.8	0/29	0	0.11.7
	Other countries	3/17	17.6	0.4-12.3	2/29	8.8	3.0-23.0
**Presence of a youth clinic in municipality**	Yes	20/307	6.5	4.3-9.9	38/589	6.5	4.7-8.7
	No	5/15	33.3	15.2-58.3	1/50	2.0	0.4-10.5
	Do not know	6/283	2.1	1.0-4.6	15/291	5.2	3.2-8.3
**Using youth clinic services**	Yes	2/66	3.0	0.8-10.4	23/312	7.4	5.0-10.8
	No	29/539	5.4	3.8-7.6	31/618	5.0	3.6-7.0
**Consulted a family doctor on STI**	Yes	14/191	7.3	4.4-11.9	33/514	6.4	4.6-8.9
	No	17/414	4.1	2.6-6.5	21/416	5.0	3.3-7,6
**Number of partners during last year**	0	1/24	4.2	0.7-20.2	0/14	0	0-21.5
	1	2/303	0.7	0.2-2.4	15/563	2.7	1.6-4.4
	2-3	11/154	7.1	4.0-12.3	19/222	8.6	5.6-13.0
	4-5	9/66	13.6	7.3-23.9	12/83	14.5	8.5-23.6
	5+	8/58	13.8	7.2-24.9	8/48	16.7	8.7-29.6
**Total number of partners**	1	0/114	0	0-3.3	0/184	0	0-2.1
	2-3	3/130	2.3	0.8-6.6	5/212	2.4	1.0-5.4
	4-5	1/103	1.0	0.2-5.3	5/157	3.2	1.4-7.2
	6-10	11/124	8.9	5.0-15.2	23/215	10.7	7.2-15.5
	10+	16/134	11.9	7.5-18.5	21/162	13.0	8.6-19.0
**Previoulsy tested for chlamydia**	Yes	11/161	6.8	3.9-11.8	35/510	6.9	5.0-9.4
	No	20/444	4.5	2.9-6.9	19/420	4.5	2.9-7.0
**Previously diagnosed with chlamydia**	Yes	4/57	7.0	2.8-16.7	19/152	12.5	8.2-18.7
	No	27/548	4.9	3.4-7.1	35/778	4.5	3.5-6.2
**Previoulsy diagnosed with genital warts**	Yes	3/40	7.5	2.6-19.9	1/64	1.6	0.3-8.3
	No	28/565	5.0	3.5-7.1	53/866	6.1	4.7-7.9
**Previously diagnosed with genital herpes**	Yes	2/2	100	0.34-100	0/16	0	0-19.4
	No	29/603	4.8	3.4-6.8	54/914	5.9	4.6-7.6
**Presence of symptoms**	Yes	10/83	12.0	6.7-20.8	20/329	6.1	4.0-9.2
	No	21/522	4.0	2.7-6.1	34/601	5.7	4.1-7.8
**Sex with a person of the same sex**	Yes	0/28	0	0-12.1	5/65	7.7	3.3-16.8
	No	31/577	5.4	3.8-7.5	49/865	5.7	4.3-7.4
**Method with casual partner**	No contraception	12/136	8.8	5.1-14.8	10/99	10.1	5.6-17.6
	Condom	7/135	5.2	2.5-10.3	9/134	6.7	3.6-12.2
	Hormonal	8/135	5.9	3.0-11.3	29/345	8.4	5.9-11.8
	Other	4/199	2.0	0.8-5.0	6/352	1.7	0.8-3.7
**Method with long-term partner**	No contraception	8/114	7.0	3.6-13.2	11/129	8.5	4.8-14.6
	Condom	4/86	4.7	1.8-11.4	1/83	1.2	0.2-6.5
	Hormonal	15/331	4.5	2.8-7.3	34/588	5.8	4.2-8.0
	Other	4/74	5.4	2.1-13.1	8/130	6.2	3.2-11.7
**Constant partner**	Yes	8/297	2.7	1.4-5.2	22/520	4.2	2.8-6.3
	No	23/308	7.5	5.0-11.0	32/410	7.8	5.6-10.8

### Sexual behaviour, use of health care services and self reported sexually transmitted diseases

Mean age for sexual debut for women was 16.7 years (range12-24) and for men 17.2 years (range 11–25). Women reported having their first sexual intercourse 6.5 (95% CI 4.0-9.0) months earlier than men (t _(1507)_ = 5.09, p < 0.001). More than half of the participants had a steady partner at the time of the survey. Close to sixty percent reported having had no more than one sexual partner during the last year while 17% reported four sexual partners or more for the same period. Men had more sexual partners during the last year than women (*χ*_(1)_^2^ =10.99, p for linear trend <0.001) although no difference in lifetime numbers of partners was observed (*χ*_(1)_^2^ =1.85, p for linear trend =0.174). Only 17,4% reported having used a condom at last sexual intercourse with a casual partner, 31% used hormonal contraceptives and 15% used no contraceptive methods (Table 1).

More women than men had ever consulted the doctor to get tested for an STI (55% vs 32%; *χ*_(1)_^2^ =6.85, p <0.001). 37 percent answered “do not know” to the question whether there was a youth clinic in their municipality. Women were more aware about the youth clinics than men (*χ*_(1)_^2^ =40.7, p <0.001), and were also more likely to use the youth clinics (women 34%, men 11%, *χ*_(1)_^2^ = 101.22, p <0.001). Women were more likely than men to previously have been tested (women 54%, men 27% *χ*_(1)_^2^ =118.70, p <0.001) and diagnosed with Chlamydia infection (women 16,2% and men 9,4%) *χ*_(1)_^2^ =14.95, p <0.001). 7% reported a history with genital warts, no gender difference (Table 1).

### Prevalence of chlamydia and factors associated with disease

The chlamydia prevalence was 5.5% (85/1535, 95% CI 4.5-6.8)), 5.8% among women (54/930, 95% CI) and 5.1% among men (31/605, 95% CI 3.8-6.8). In the age group 18–21 the chlamydia prevalence was 6.5% (46/707, 95% CI 4.9-8.6) and in the age group 22–25 4.7% (39/828, CI 3.5-6.3). The chlamydia prevalence was 5.5% (80/1451, 95% CI 4.5-6.8) among Norwegian born and 0% among participants born in Europe and North America. Among the participants from other regions the chlamydia prevalence was 11.9% (5/42, 95% CI 5.2-25.0). The five chlamydia cases came from Asia (2) South and Central America(2) and Oceania (1).

In a multivariate analysis the following variables remained associated with chlamydia infections: For men a greater number of partners during the last year increased the odds of infection (p < 0.001), and not having a local youth clinic present increased the odds of infection by 8.6 times (OR 8.6, 95% CI 2.2-33.9) compared to those who reported having a local youth clinic in their home municipality. Only 15 reported not having a local youth clinic. For women a greater number of partners during the last year was associated with increased odds of infection (p < 0.001) while having no previous chlamydia diagnosis decreased the odds of infection compared to those who had been diagnosed with chlamydia before(OR 0.3, 95% CI 0.2-0.7). Those who had not previously consulted a family doctor for STIs had 2.1 (95% CI 1.1-4.2) higher odds of infection compared to those who had previously consulted their family doctor for STIs (Table 2).

**Table 2:** Factors associated with genital chlamydia infection among men and women aged 18–25 years in Rogaland, Norway

**Sample characteristic**	**n/N**	**Crude OR**	**95%CI**	*Adjusted OR	**95% CI**	**p-value**
**Men**						
Presence of a local youth clinic						<0.001
Yes	20/307	1	Reference	1	Reference	
No	5/15	7.2	2.2-23.0	8.6	2.2-33.5	
Do not know	6/283	0.3	0.1-0.8	0.3	0.1-0.7	
Using youth clinic’s services						0.066
Yes	2/66	1	Reference	1	Reference	
No	29/539	1.8	0.4-7.8	4.1	0.9-19.1	
Number of partners during last year						<0.001
0	1/24	6.5	0.6-74.9	6.2	0.5-78.2	
1	2/303	1	Reference	1	Reference	
2-3	11/154	11.6	2.5-52.9	11.2	2.4-52.5	
4-5	9/66	23.8	5.0-112.9	28.6	5.8-142.0	
More than 5	8/58	24.1	5.0-116.7	30.5	6.0-155.5	
**Women**						
Previously diagnosed with Chlamydia						
Yes	19/152	1	Reference	1	Reference	<0.001
No	35/778	0.3	0.2-0.6	0.3	0.2-0.6	
Previously diagnosed with genital warts						
Yes	1/64	1	Reference	1	Reference	0.071
No	53/866	4.1	0.6-20.2	6.5	0.8-49.1	
Consulting family doctor on STI						
Yes	18/352	1	Reference	1	Reference	0.027
No	36/578	1.2	0.7-2.2	2.1	1.1-4.2	
Number of partners during last year						
0	0/15	-	-	-	-	<0.001
1	15/563	1	Reference	1	Reference	
2-3	19/222	3.4	1.7-6.9	3.2	1.6-6.5	
4-5	12/83	6.2	2.8-13.7	6.5	2.8-14.7	
More than 5	8/48	7.3	2.9-18.3	7.9	3.0-20.6	

Estimated in multiple logistic regression. N = 1535.'

*adjusted odds ratios are adjusted for the variables in the table as a result of stepwise procedure, backwards elimination.

## Discussion

This study is the only population based study measuring chlamydia and associated factors in young Norwegian men and women in one county where the study sample is drawn from the national population register, which includes the entire population, and not from a selected group of individuals. It is also among the rare prevalence studies were home sampling by postal recruitment was used. We found a chlamydia prevalence of 5.5% in our population. The likelihood of infection increased with a greater number of partners. Among women, those with a previous chlamydia diagnosis (and those who had not previously consulted their family doctor on STIs) were more likely to have a current infection. Among men the odds of infection increased by 8.6 times if they believed there was no local youth clinic present. The majority did not use condom at last sexual intercourse with a casual partner.

### Prevalence of chlamydia, and other self reported STIs

We measured a chlamydia prevalence of 5.5%. Other chlamydia prevalence surveys in Norway have found from 1 to 9% depending on the basis of their population.[7,8][9-13]. A study among all high school graduates (18 year olds) in one Norwegian municipality found a chlamydia prevalence of 2% among those who had had their sexual debut [9]. Another prevalence study among Norwegian male students aged 18–30 recruited at student campus and students health centres measured a chlamydia prevalence of 7.8% [10]. A home based chlamydia prevalence survey among patients 18–29 of age listed at a general practice in Oslo gave a chlamydia prevalence of 3% [7]. A chlamydia prevalence of 9% was found among young girls 16–19 years seeking youth clinic for hormonal prevention [14] and 6% among women (16–24) seeking abortion [15]. Our results are in the mid-range of what is found in other publications from Norway, lower than in the populations seeking health care, but higher than most other studies conducted in different populations outside the health care system. Population based studies offering home sampling for chlamydia outside Norway has shown a prevalence ranging from 1 to 7%[16][17-20].

Seven percent reported a history of genital warts in our sample. This is lower than reported from other Nordic countries.[21].

### Sexual behaviour and health care use

According to the national sexual behaviour studies in Norway the age for sexual debut has been decreasing from 1987 to 2002 when the median age was 17,5 years for men and 17.1 years for women [22]. Another publication on decreasing trends for age for sexual debut among Norwegian youth from the same time-period reported median age for sexual debut to 18.0 years for men and 16.7 years for women in 2002 [23] In the study among male students the median age for sexual debut was 17.5[10]. A recent publication on sexual behaviour among Nordic women reported a median age of 16 years for the first intercourse for the Norwegian women involved [24]. Our results do not differ much from these other studies. We found a slightly lower mean age for sexual debut among the men in our study. This could be the result of a continuous decreasing trend. However, it could also indicate that men with an earlier sexual debut or who felt more at risk of infection were more likely to take part in our study. Only 17% reported using condom at last sexual intercourse with casual partner. This result is supported by the National sexual behaviour studies[22]. In the study among male students 76% reported not using condom at last intercourse[10], The condom use among young Norwegians is however less frequent than reported from other European countries [25,26].

### Factors associated with infection

A greater number of partners is well documented as a risk factor for chlamydia[26-28]. Condom use[26,29] and presence of genital symptoms[30] have by many been documented as risk factors, but this is not universal. We also found that having multiple partners was associated with a positive test result, but condom use at last sexual intercourse with non- steady partner and presence of genital symptoms (discharge, endocervical bleeding, pelvic pain, urethral itching, dysuria) was not associated with infection, neither was any of the socio-demographic factors measured in this study (age, marital status, education, occupation, country of birth).

Among men, reporting that the home municipality lacked a youth clinic was associated with chlamydia, indicating that youth clinics may be a valuable contribution to lower the community prevalence of chlamydia among men. This is probably an indirect effect of diagnosing and treating young women as these are the overwhelming majority of users of these drop-in facilities that offer condoms and chlamydia tests free of charge for young people up to the age of 20 years. In Rogaland county, the majority of the municipalities do offer a local youth clinic. Perhaps also the mere visibility of such a clinic can contribute to a more protective behaviour among men. The finding should, however, be interpreted with caution because of the small group size reporting not having a local youth clinic in their home municipality.

Close to half of the men in our survey did not know if there was a youth clinic present in their municipality. This group had a lower chlamydia prevalence, only 2.1%. We may speculate that these men have less sexual risk behaviour and therefore never have felt the need to find out about youth clinics in their area. Alternatively, these men were more likely to take part in the study and use our mailed testing kits since they were not aware of the youth clinic with testing facilities nearby.

Among women, we found that having had chlamydia diagnosed previously was associated with an increased risk of current infection. This is consistent with findings from a substantial body of previous literature [31,32]. 63% of the chlamydia positive women with a previous chlamydia diagnosis in our study had changed their sexual partner within the last 6 months. The temporal association between the previous infection and this positive test is, however, unknown. We can therefore not establish whether the positive test result represents treatment failure, lack of partner treatment or a new infection with a new sexual partner. Many factors could contribute to this result. Our results may indicate that sexual risk behaviour is persistent and that a chlamydia diagnosis may not lead to behavioural change. It highlights the need for guidance on safe sexual behaviour at the time of treatment, more careful partner tracing and treatment and a special need to target women with a previous chlamydia diagnosis in screening. On the other hand, women who had consulted their family doctor on STIs were less likely to have a current infection than those who had not consulted their family doctor. Possible explanations could be that women who consult their family doctor for STIs are those with less sexual risk behaviour or that they in fact have received good guidance on safe sexual behaviour from their doctor.

### Limitations

This cross sectional study was part of a clinical trial of a postal screening system for chlamydia in Norway. Only those who wanted to have a chlamydia test taken answered the questionnaire. The response rate is therefore lower than what would have been expected if the participants would only have to answer a questionnaire. The uptake of the screening offer was also lower than anticipated and what has been the case in similar studies[16-18]. The low response rate raises the concern of a potential selection bias in our results. It is a cause for concern regarding the validity of the prevalence estimates. As we have no information on the non-responders, including their chlamydia status, we will not know if reasons not to participate in the study are the same as factors associated with infection. However, regarding the association between chlamydia and risk factors within the group of respondents, the overall response rate is less of a concern. The low response rate would lead to biased associations only if participation was associated with *both* chlamydia *and* the risk factors in question, which is more unlikely.

The rather low number of participants and thereby chlamydia cases in our study gives the study limited power to detect associations between infection and exposures. We can therefore not exclude other associations than those detected by our study.

Questionnaires were filled out at the same time as the home sample was submitted. With this cross sectional study design we are not allowed to determine a causative effect of the different factors associated with infection. The relatively long recall period could cause misreporting of own risk behaviour. However, differential misclassification is avoided since infectious status was not known at the time the participants fill out the questionnaires.

On the other hand, by using postal recruitment and allowing participants to take the chlamydia test at home, we might have reached a broader part of the population than those seeking the health care service believing they are at risk.

### Conclusion and recommendations

Young Norwegian men and women have their sexual debut at an early age and the majority do not use condom when having casual sex. We found a chlamydia prevalence of 5.5% in our population. The probability of infection increased with a greater number of partners, and among men the odds of infection increased if their home municipality lacked a youth clinic. For women having a previous chlamydia diagnosis increased the probability of infection while having previously consulted their family doctor decreased the odds of infection.

Only a very low proportion of the men in our sample had ever visited their local youth clinic. Our results indicate the importance of having a visible youth clinic in each municipality. It also suggests targeting women with a previous chlamydia diagnosis.

## Competing interests

The authors declare that they have no conflict of interest, financial or non - financial. The research was funded by the Norwegian Institute of Public Health.

## Authors’ contributions

HK has planned and designed the research study, been involved in the acquisition of data, done the analysis of and interpretation of data and drafted and revised the manuscript. AG has made a substantial contribution to the analysis and interpretation of data and in drafting and revising the manuscript critically. PAa has been involved in the planning and design of the research study, participated in the analysis and interpretation of data and has made a substantial contribution to the draft and revision of the manuscript. All authors have read and approved the final manuscript.

## Pre-publication history

The pre-publication history for this paper can be accessed here:

http://www.biomedcentral.com/1471-2334/12/150/prepub
